# Flavonoids as Vasorelaxant Agents: Synthesis, Biological Evaluation and Quantitative Structure Activities Relationship (QSAR) Studies

**DOI:** 10.3390/molecules16108257

**Published:** 2011-09-28

**Authors:** Xiaowu Dong, Yanming Wang, Tao Liu, Peng Wu, Jiadi Gao, Jianchao Xu, Bo Yang, Yongzhou Hu

**Affiliations:** 1 ZJU-ENS Joint Laboratory of Medicinal Chemistry, College of Pharmaceutical Sciences, Zhejiang University, Hangzhou 310058, Zhejiang, China; 2 Institute of Pharmacology & Toxicology, College of Pharmaceutical Sciences, Zhejiang University, Hangzhou 310058, Zhejiang, China

**Keywords:** flavonoids, molecular descriptors, vasorelaxant agents, qsar, erm-mlr

## Abstract

A series of 2-(2-diethylamino)-ethoxychalcone and 6-prenyl(or its isomers)-flavanones **10a,b** and **11a**–**g** were synthesized and evaluated for their vasorelaxant activities against rat aorta rings pretreated with 1 μM phenylephrine (PE). Several compounds showed potent vasorelaxant activities. Compound **10a** (EC_50_ = 7.6 μM, *E_max_* = 93.1%), the most potent one, would be a promising structural template for development of novel and more efficient vasodilators. Further, 2D-QSAR analysis of compounds **10a,b** and **11c-e** as well as thirty previously synthesized flavonoids **1-3** and **12-38** using Enhanced Replacement Method-Multiple Linear Regression (ERM-MLR) was further performed based on an optimal set of molecular descriptors (H5m, SIC2, DISPe, Mor03u and L3m), leading to a reliable model with good predictive ability (*R_train_^2^* = 0.839, *Q_loo_*^2^ = 0.733 and *R*_test_^2^ = 0.804). The results provide good insights into the structure- activity relationships of the target compounds.

## 1. Introduction

**Figure 1:**
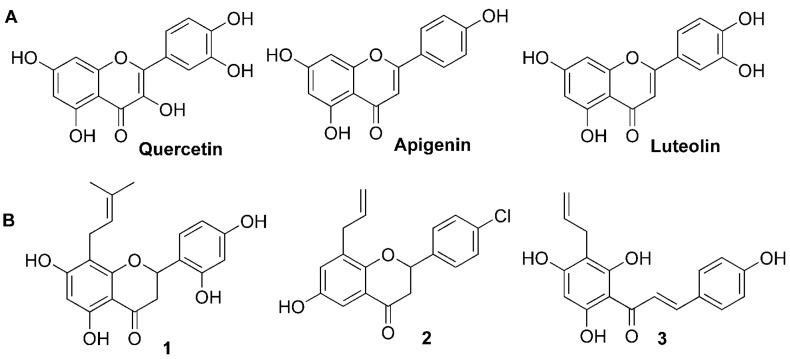
The 2D structure of the flavonoids with potent vasorelaxant activities.

Flavonoids, a class of plant secondary metabolites, are polyphenols based around a phenylbenzopyrone structure [1,2]. According to their different skeletons, they are categorized into flavones, flavanones, chalcones, flavonols, isoflavones and aurones, *etc.* [3]. Associations of dietary flavonoid intake with lower incidence of cardiovascular disease have been reported in several epidemiological studies [4,5,6,7]. Since one of the main causes of cardiovascular diseases is the involvement of increasing tonicity or loss of relaxation capacity of vascular tissues, vasodilators are a beneficial treatment for cardiovascular diseases. Until now, a large number of flavonoids such as quercetin, luteolin and apegenin (Figure 1A) have been found to show vasorelaxant activities [8,9,10]. In addition, flavonoids can reduce the superoxide levels of vascular endothelium under oxidative stress conditions and improve endothelial cell disfunction, which is also crucial for treatment of cardiovascular diseases [11,12].

Previously, some quercetin analogues were synthesized by our group and evaluated for their vasorelaxant activities, the results of which indicated that the LogP values of the synthesized flavonoids were correlated with their vasorelaxant activities [13]. In order to further investigate the effect of lipophilic change on vasorelaxant activity, the prenyl (or allyl) group was introduced into various flavonoid scaffolds (e.g., chalcones, flavanones, flavones and aurones). Some of them exhibited potent vasorelaxant activity, such as 8-prenyl (or allyl)-flavanone derivatives **1**, **2** and chalcone derivative **3** (Figure 1B; the EC_50_ values of **1**, **2** and **3** are 9.3, 4.6 and 24.0 μM, respectively) [14,15,16]. These results prompted us to discover more potent lipophilic flavonoid derivatives and investigate the comprehensive structure-activity relationship of these compounds.

**Figure 2:**
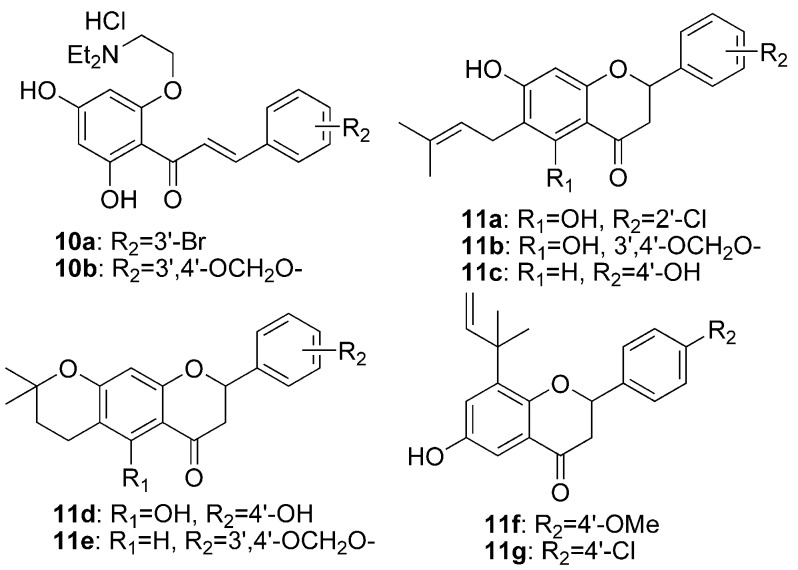
The structures of the target flavonoids synthesized in this study.

In this study, 6-prenyl(or its isomers)-flavanones and 2-(2-diethylamino)-ethoxychalcone derivatives **10a,b** and **11a**–**g** (Figure 2) were prepared, considering the effect of prenyl(or its isomers) in the C-6 position of flavanones as well as the introduction of 2-(diethylamino)ethyl groups in chalcones. The vasorelaxant activities of the synthesized flavonoids were assayed on rat-aorta rings pretreated with 1 μM phenylephrine (PE). Furthermore, Enhanced Replacement Method-Multiple Linear Regression (ERM-MLR) was applied to select the most optimal set of molecular descriptors and set up a linear model to probe the quantitative structure-activity relationships (QSAR) of the target compounds.

## 2. Results and Discussion

### 2.1. Chemistry

**Scheme 1:**
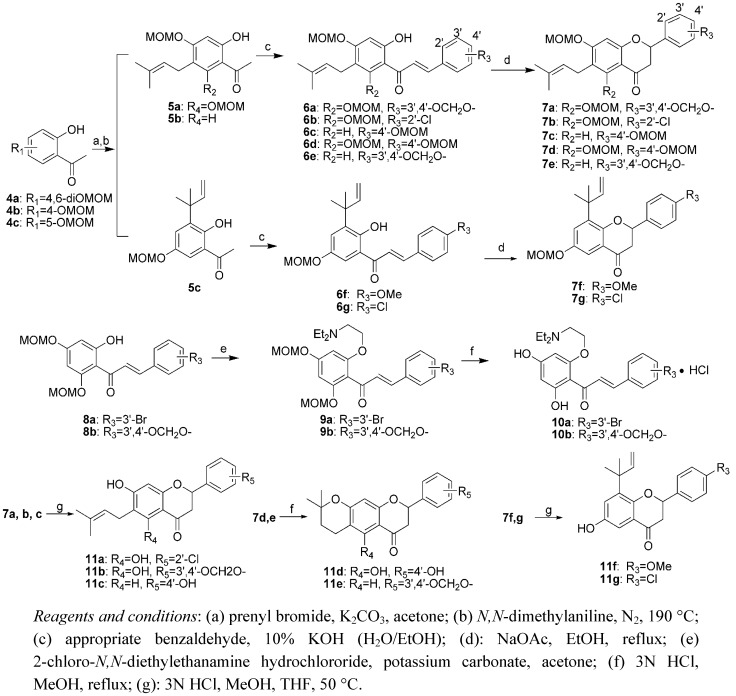
Synthesis of prenylflavonoids **10a,b** and **11a**–**g**.

The synthetic pathway to the nine prenylflavonoids **10a,b** and **11a**–**g** is outlined in Scheme 1. Acetophenone **4** was allylated with prenyl bromide and successively heated at 220 °C to afford Claisen rearrangement products **5**. Condensation of **5** with the corresponding benzaldehydes in aqueous alcoholic alkali at room temperature afforded chalcones **6**. Cyclization of **6** in a solution of sodium acetate in ethanol under reflux conditions gave flavanone **7**. Compound **9** was obtained by the treatment of chalcone **8** with 2-chloro-*N,N*-diethylethanamine hydrochloride in the presence of potassium carbonate in acetone. Deprotection of the methoxymethyl groups of **7a**-**c**, **7f**, **7g** and **9a,b** were carried out in 3N HCl/MeOH/THF (2:5:5, v/v) to give the expected products **11a**-**c**, **11f**, **11g** and **10a,b**, while demethoxymethylation of compounds **7d** and **7e** in 3N HCl/MeOH (1/1, v/v) was observed to afford dihydropyranoflavones **11d** and **11e** bearing cyclic prenyl groups. The structures of all the synthesized compounds were elucidated by ^1^H-NMR and ESI-MS.

### 2.2. Vasorelaxant Activity and SAR

**Figure 3:**
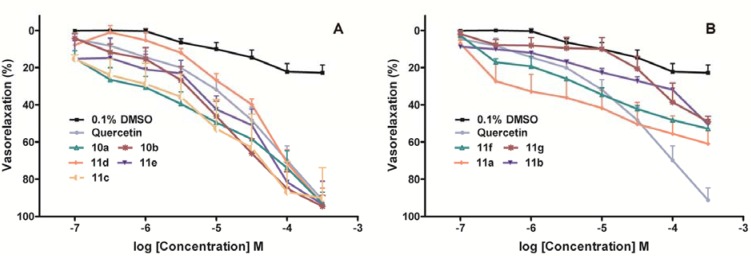
Effects of flavonoids on relaxation in aortic rings with endothelium pre-contracted with 1 μM phenylephrine. Flavonoids were added cumulatively to achieve the appropriate concentrations. Results are expressed as means ± standard error of mean in terms of percentage relaxation of the contraction to PE (n = 3 ~ 4). (A) Flavonoids with highly vasorelaxation effect; (B) Flavonoids with weakly vasorelaxation effect.

Vasorelaxant activities of compounds **10a**,**b** and **11a-g** were evaluated against aortic rings with endothelium pre-contracted with 1 μM phenylephrine. The promoted relaxation of all the compounds was dose-dependent manner, with the maximal effect observed at 300 μM. Several compounds showed more potent vasorelaxation than the positive control quercetin based on the result of either *E_max_* or EC_50_. As shown in Table 1, flavonoids **10a,b** and **11c**-**e** inducing 50% relaxation at small concentration (EC_50_ < 100 μM) with good efficacy (*E_max_* > 90%) were considered to be good relaxing agents, while the remaining compounds **11a,b,f** and **11g** were regarded as weak vasodilators (EC_50_ > 100 μM; *E_max_* < 70%). Concentration–relaxation curves of the flavonoids in two categories are shown in Figure 3. The effects on vasorelaxant activities of prenyl (or its isomer) on C-6 of flavonoids were investigated, showing that the introduction of a cyclic prenyl group resulted in good vasorelaxant activity, as exemplified in dihydropyranoflavones **11d** and **11e** (**11d**: EC_50_ = 78.7 μM, *E_max_* = 93.5%; **11e**: EC_50_ = 53.5 μM, *E_max_* = 93.6%). The introduction of a 6-prenyl or 8-(1,1-dimethyl)allyl group on A ring of flavanone (e.g., **11a,b,f** and **11g**) led to the weak to moderate activity, except for compound **11c**. The 2-(2-diethylamino)ethoxychalcone derivatives **10a,b** showed better vasorelaxant activities (EC_50_ of **10a** and **10b** were 7.6 and 13.7 μM, respectively), indicating that replacement of prenyl with a 2-(diethylamino)ethyl group on chalcone skeletons retained the potent activity.

**Table 1:** The vasorelaxant activities of flavonoids **10a**,**b** and **11a-g**, in rat aorta rings pre-contracted with PE.

Compd.	EC_50_(μM)	*E_max_*(%)	Compd.	EC_50_(μM)	*E_max_*(%)
Quercetin	244	91.3	**11c**	19.9	90.3
**10a**	7.6	93.1	**11d**	78.7	93.5
**10b**	13.7	99.5	**11e**	53.5	93.6
**11a**	N.D.	66.2	**11f**	N.D.	52.9
**11b**	N.D.	50.6	**11g**	N.D.	49.1

### 2.3. Development of QSAR Model Using ERM-MLR

For a quantitative understanding of the structure-vasorelaxant activities relationship of the flavonoids, ERM-MLR was applied to set up a linear QSAR model to explore the EC_50_ of the newly synthesized five flavonoids **10a,b** and **11c-e** with efficacious EC_50_ and the previously reported thirty flavonoids **1-3** and **12**-**38** [14,15,16,19]. At first, the calculated molecular descriptors that stayed constant for all molecules were eliminated and one of each correlated pair was deleted according to the results of correlation analysis, resulting in a pool of 299 descriptors for further QSAR model development. Then, ERM-MLR was applied to select an optimal set of molecular descriptors and four QSAR models with different number of variables (n = 2~5) were obtained considering that the number of variables selected should be kept lower than 20 percent of the number of coupounds in training set in avoidance of overlay problem. Commonly, the higher values of correlation coefficient (*R*^2^) for both training and test set, leave-one-out (loo) cross correlation coefficient (*Q*_loo_^2^) and the lower standard deviation value (SD), the better the results. Thus, five molecular descriptors in ERM-MLR model has an optimal influence on improving correlation (Table 2). The physical-chemical meanings of the molecular descriptors in the best model are listed in Table 3, forming the equation as given in Equation (1):

−Log(EC_50_) = 0.442*H5m − 0.465*SIC2 + 0.287*DISPe − 0.518*Mor03u − 0.574*L3m + 4.376 (1)

**Table 2:** The statistical parameters of obtained ERM-MLR models.

No.			ERM-MLR		n ^a^
	*R_train_^2^*	*Q_loo_^2^*		*R_test_^2^*	
1	0.603	0.483		0.366	n = 2
2	0.734	0.648		0.336	n = 3
3	0.786	0.705		0.527	n = 4
4	0.839	0.733		0.804	n = 5

^a^ The number of molecular descriptors selected in ERM-MLR QSAR models.

**Table 3:** The molecular descriptors involved in the ERM-MLR models and their corresponding definition.

Symbol	Class	Definition
H5m	GETAWAY descriptors	H autocorrelation of lag 5/weighted by atomic masses
SIC2	information indices	structural information content (neighborhood symmetry of
		2-order) information indices
DISPe	geometrical descriptors	d COMMA2 value/weighted by atomic Sanderson
		electronegativities
Mor03u	3D-MoRSE descriptors	Mor03u 3D-MoRSE - signal 03/unweighted
L3m	WHIM descriptors	3rd component size directional WHIM index/weighted by
		atomic masses

**Figure 4:**
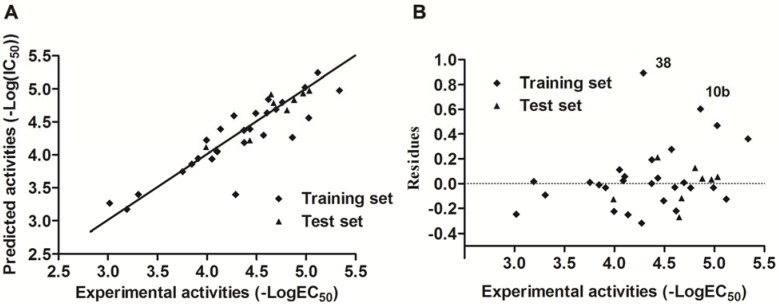
Predicted *vs.* experimental vasorelaxant activities (−LogEC_50_) of the QSAR model developed by EMR-MLR.

The quality of the fit for the resulting model [Equation (1)] can be judged by the determination coefficient (*R_train_^2^* = 0.839), the standard deviation (SD = 0.263) and the Fishers estimate of statistical significance value (F = 21.9). Its predictive ability is characterized by leave-one cross validation (Q_loo_^2^ = 0.733, SD = 0.303) and test set validation (*R*_test_^2^ = 0.804, SD = 0.145). The predictions provided by Equation (1) are shown in Table 4. In Figure 4A the predictions in function of the experimental coefficients are plotted, suggesting that the thirty-five data points (overall data set) follow a straight line trend. In addition, Figure 4B shows the graph between the residuals *vs.* experimental vasorelaxant activity values. The lower residuals obtained in both the training and test set of compounds in the model indicated that the developed model are valuable and has the capability to establish the relationship between the structure and vasorelaxant activity for flavonoids used in this study. For example, the vasorelaxant activities of the highly potent compound **10a** and **11d** were accurately predicted using the established QSAR model. The values of H5m, SIC2, DISPe, Mor03u and L3m are optimal for Equation(1), contributing to the fact that the prediction activity is as good as its actual activity (predictive –log(EC_50_) of **10a**: 5.245; actual −log(EC_50_) of **10a**: 5.119), while the unfavorable values of H5m, SIC2, DISPe, Mor03u and L3m led to the lower prediction activity compared with compound **10a** but close to its actual activity (predictive –log(EC_50_) of **11d**: 4.048; actual −log(EC_50_) of **11d**: 4.104). However, inaccurate prediction of several compounds was also observed. As exemplified by compound **38**, the weak prediction [predictive –log(EC_50_) of **38**: 3.399] may due to the weak activities exhibited by flavone skeleton compounds [actual −log(EC_50_) of compounds **33**~**37**: <4.088], but compound **38** actually exhibited moderate activity [actual −log(EC_50_) of **38**: 4.290], resulting in the worst fit of **38** (residual: 0.891). In fact, this problem would be fixed in further study, accompanied that more vasorelaxant flavonoids were identified and applied in improving the model.

**Table 4:** The experimental and prediction vasorelaxant activities of flavonoids **1-3**, **10a**,**b**, **11c-e** and **12-38** used in the established QSAR model. 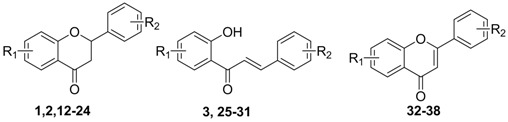

Cmpd.	R_1_	R_2_	Exp. activities		Pred.	Res.	Ref.
			EC_50_(μM)	*p*(EC_50_)	*p*EC_50_(μM)		
**1 ^a^**	5,7-diOH-8-prenyl	2',4'-diOH	9.3	5.032	4.977	0.055	[16]
**2**	6-OH-8-allyl	4'-Cl	4.6	5.337	4.976	0.361	[14]
**3**	3-allyl-4,6-diOH	4'-OH	24.0	4.620	4.841	−0.221	[15]
**10a**	/	/	7.6	5.119	5.245	−0.126	/
**10b**	/	/	13.7	4.863	4.260	0.603	/
**11c**	/	/	19.9	4.762	4.796	−0.034	/
**11d**	/	/	78.7	4.104	4.048	0.056	/
**11e**	/	/	53.5	4.272	4.592	−0.32	/
**12**	5,7-diOH	3'-Br	42.4	4.373	4.372	0.001	[14]
**13**	5,7-diOH	3',4'-OCH_2_O-	20.1	4.697	4.688	0.009	[14]
**14**	6-OH-8-allyl	4'-OH	34.5	4.991	5.024	−0.033	[14]
**15^a^**	5,7-diOH-8-allyl	3'-OH	37.0	4.432	4.219	0.213	[14]
**16**	5,7-diOH-8-allyl	4'-OH	32.1	4.493	4.632	−0.139	[14]
**17**	7-OH-8-allyl	3',4'-OCH_2_O-	9.4	5.027	4.558	0.469	[14]
**18**	7-OH-8-allyl	4'-OH	26.9	4.570	4.295	0.275	[14]
**19**	7-OH-8-allyl	4'-Cl	142	3.848	3.858	−0.01	[14]
**20^a^**	5,7-diOH-8-prenyl	3',4'-OCH_2_O-	13.2	4.879	4.837	0.042	[19]
**21**	5,7-diOH-8-prenyl	3',4',5'-triMeO-	24.8	4.606	4.636	−0.03	[19]
**22**	5,7-diOH-8-prenyl	3'-MeO-4'-OH	101	3.996	4.221	−0.225	[19]
**23^a^**	5,7-diOH-8-prenyl	3'-Br	15.6	4.807	4.680	0.459	[19]
**24**	5,7-diOH-8-prenyl	4'-OH	72.7	4.138	4.389	−0.251	[16]
**25^a^**	4,6-diOH	3'-Br	21.3	4.672	4.789	−0.117	[14]
**26^a^**	4,6-diOH	3',4'-OCH_2_O-	22.5	4.648	4.919	−0.271	[14]
**27**	3-allyl-4-OH	4'-OH	89.1	4.050	3.937	0.113	[14]
**28**	3-prenyl-4,6-diOH	3',4'-OCH_2_O-	42.1	4.376	4.184	0.192	[19]
**29^a^**	3-prenyl-4-OH	4'-OH	102	3.991	4.116	−0.125	[15]
**30**	4-OH-5-prenyl	3'-OH	123	3.910	3.943	−0.033	[15]
**31^a^**	4-OH-5-prenyl	2', 4'-diOH	10.7	4.971	4.937	0.034	[15]
**32**	5,7-diOH	3'-Br	36.8	4.434	4.389	0.045	[14]
**33**	7-OH	3',4'-OCH_2_O-	81.7	4.088	4.065	0.023	[14]
**34**	5,7-diOH-8-prenyl	3'-Br	959	3.018	3.266	−0.248	[19]
**35**	5,7-diOH-8-prenyl	3',4',5'-triMeO-	643	3.192	3.175	0.017	[19]
**36**	5,7-diOH-8-prenyl	3'-MeO-4'-OH	493	3.307	3.399	−0.092	[19]
**37**	5,7-diOH-8-prenyl	2',4'-diOH	176	3.754	3.744	0.01	[16]
**38**	6-OH-8-allyl	4'-OH	51.3	4.290	3.399	0.891	[14]

^a^ The compounds were used as test set.

## 3. Conclusions

In this study, some synthesized flavonoid derivatives were characterized as agents with remarkable vasorelaxant activities. The preliminary structure-activity relationships studies revealed that cyclic prenyl and 2-(diethylamino)ethyl groups are beneficial for vasorelaxant activity. 2D-QSAR analysis using ERM-MLR was performed to set up a statistically reliable model with good predictive ability (*R_train_^2^* = 0.839, Q_loo_^2^ = 0.733 and *R*_test_^2^ = 0.804), and five descriptors (H5m, SIC2, DISPe, Mor03u and L3m) were found to be closely correlated with the observed vasorelaxant activities of the target compounds.

## 4. Experimental

### 4.1. General

Melting points were measured on a Büchi B-540 apparatus and are uncorrected. All ^1^H-NMR spectra were recorded on a 400 MHz spectrometer (Brüker AM). Chemical shifts were expressed as *δ* values in parts in million (ppm) relative to tetramethylsilane (TMS). Mass spectral data were obtained on an Esquire-LC-00075 spectrometer. Reagents and solvents were purchased from common commercial suppliers and were used without further purification. Compounds **4a-c**, **6c**, **7c**, **8a,b** and **11c** were prepared according to the procedures reported in previous references [14,17].

### 4.2. General Method for Synthesis of Prenylated Acetophenones ***5***

A solution of compound **4**, prenyl bromide and potassium carbonate in DMF (100 mL) was heated at 100 °C under a N_2_ atmosphere for 6 h, and then the reaction mixture was poured into cold water and extracted with ethyl acetate. The organic phase was washed with brine and dried over anhydrous sodium sulfate. After removal of the solvent, the residue was dissolved in *N,N*-dimethylaniline (50 mL) and then heated at 190 °C for 3~5 h. The mixture was concentrated under vacuum, and the residue was purified by column chromatography on silica gel using (30/1, v/v) as eluent to give compounds **5**.

*2-Hydroxy-5-(3,3-dimethyl)allyl-4,6-dimethoxymethoxyacetophenone* (**5a**): Reagents: compound **4a** (10.0 g, 39.1 mmol), prenyl bromide (8.73 g, 58.6 mmol) and potassium carbonate (10.8 g, 78.2 mmol). Product: yellow oil (4.93 g, 39%); ^1^H-NMR (CDCl_3_, *δ*): 1.68 (s, 3H), 1.76 (s, 3H), 2.69 (s, 3H), 3.30 (d, 1H, *J* = 6.4 Hz), 3.45 (s, 3H), 3.51 (s, 3H), 4.95 (s, 2H), 5.14 (m, 1H), 5.21 (s, 2H), 6.46 (s, 1H), 12.93 (s, 1H, OH). ESI-MS: *m/z* [M-H]^−^ 323.

*2-Hydroxy-5-(3,3-dimethyl)allyl-4-dimethoxymethoxyacetophenone* (**5b**): Reagents: compound **4b** (10.0 g, 51.0 mmol), prenyl bromide (11.4 g, 76.5 mmol) and potassium carbonate (14.1 g, 102.0 mmol). Product: yellow oil (7.4 g, 55%). ^1^H-NMR (CDCl_3_, *δ*): 1.72 (s, 3H), 1.75 (s,3H), 2.55 (s, 3H), 3.25 (d, 2H, *J* = 7.2Hz), 3.47 (s, 3H), 5.23 (s, 2H), 5.26 (m, 1H), 6.60(s,1H), 7.43(s,1H), 12.54(s,1H). ESI-MS: *m/z* [M-H]^−^ 263.

*2-Hydroxy-3-(1,1-dimethyl)allyl-5-dimethoxymethoxyacetophenone* (**5c**): Reagents: compound **4c** (10.0 g, 51.0 mmol), prenyl bromide (11.4 g, 76.5 mmol) and potassium carbonate (14.1 g, 102.0 mmol). Product: yellow oil (6.19 g, 46%). ^1^H-NMR (CDCl_3_, *δ*): 1.44 (s, 3H), 1.47 (s, 3H), 2.55 (s, 3H), 3.48 (s, 3H), 4.85 (d, 1H, *J* = 17.2 Hz), 4.93 (d, 1H, *J* = 10.8 Hz), 5.24 (s, 2H), 6.05 (dd, 1H, *J* = 10.8, 17.2 Hz), 7.06 (d, 1H, *J* = 2.0 Hz), 7.33 (d, 1H, *J* = 2.0 Hz), 12.87 (s, 1H, OH). ESI-MS: *m/z* [M-H]^−^ 263.

### 4.3. General Method for Synthesis of Prenylated Chalcones ***6***

To a cold solution of the acetophenone **5** and appropriate benzaldehyde in H_2_O-EtOH (1/4, v/v, 3 mL), 20% KOH in H_2_O-EtOH (1/4, v/v, 3 mL) was added with stirring. The resulting mixture was stirred under nitrogen at room temperature for 36 h, and then poured into ice-water (50 mL). The solution was acidified to pH ~ 2 with 1 N HCl, and extracted with ethyl acetate (20 mL × 3 times). The organic phase was washed with brine, dried over anhydrous sodium sulfate, and concentrated *in vacuo*. The residue was purified by column chromatography on silica gel using petroleum ether-ethyl acetate as eluant to give the desired compound **6**.

*2-Hydoxy-2'-chloro-4,6-dimethoxymethoxy-5-(3,3-dimethyl)allylchalcone* (**6a**): Reagents: compound **5a** (500.2 mg, 1.54 mmol), 2-chlorobenzaldehyde (227.8 mg, 1.62 mmol). Eluent: petroleum ether- ethyl acetate (20:1, v/v). Product: yellow oil (468.7 mg, 68%). ^1^H-NMR (CDCl_3_, *δ*): 1.68 (s, 3H,), 1.78 (s, 3H), 3.32 (d, 2H, *J* = 6.8 Hz), 3.43 (s, 3H), 3.49 (s, 3H), 5.21 (m, 1H), 5.25 (s, 2H), 5.26 (s, 2H), 6.39 (s, 1H,), 7.30 (m, 2H), 7.43 (m, 1H), 7.67 (m, 1H), 7.88 (d, 1H, *J* = 16.0 Hz), 8.12 (d, 1H, *J* = 16.0 Hz), 13.86 (s, 1H, OH). ESI-MS: *m/z* [M+H]^+^ 447.

*2-Hydoxy-3',4'-dioxymethylene-4,6-dimethoxymethoxy-5-(3,3-dimethyl)allylchalcone* (**6b**): Reagents: compound **5a** (500.0 mg, 1.54 mmol), 3,4-dioxymethylenebenzaldehyde (243.1 mg, 1.62 mmol). Eluent: petroleum ether-ethyl acetate (18:1, v/v). Product: yellow oil (443.3 mg, 63%). ^1^H-NMR (CDCl_3_, δ): 1.70 (s, 3H,), 1.74 (s, 3H), 3.31 (d, 2H, *J* = 6.8 Hz), 3.50 (s, 3H), 3.54 (s, 3H), 5.21 (m, 1H), 5.24 (s, 2H), 5.28 (s, 2H), 6.01 (s, 2H), 6.43 (s, 1H), 6.83 (d, 1H, *J* = 7.6 Hz), 7.06 (dd, 1H, *J* = 7.6, 2.0 Hz), 7.08 (d, 1H, *J* = 2.0 Hz), 7.71 (d, 1H, *J* = 16.0 Hz), 7.78 (d, 1H, *J* = 16.0 Hz), 13.90 (s, 1H). ESI-MS: *m/z* [M+H]^+^ 457.

*2-Hydoxy-4,4',6-trimethoxymethoxy-5-(3,3-dimethyl)allylchalcone* (**6d**): Reagents: compound **5a** (500.2 mg, 1.54 mmol), 3,4-dimethoxymethoxybenzaldehyde (269.1 mg, 1.62 mmol). Eluent: petroleum ether-ethyl acetate (18:1, v/v). Product: yellow oil (437.2 mg, 60%) . ^1^H-NMR (CDCl_3_, *δ*): 1.68 (s, 3H), 1.79 (s, 3H), 3.32 (d, 2H, *J* = 6.8 Hz), 3.47 (s, 3H), 3.52 (s, 3H), 3.53 (s, 3H), 5.21 (m, 1H), 5.22 (s, 2H), 5.23 (s, 2H), 5.25 (s, 2H), 6.42 (s, 1H,), 7.06 (d, 2H, *J* = 8.8 Hz), 7.55 (d, 2H, *J* = 8.8 Hz), 7.76 (d, 1H, *J* = 15.6 Hz), 7.85 (d, 1H, *J* = 15.6 Hz), 13.82 (s, 1H). ESI-MS: *m/z* [M+H]^+^ 473.

*2-Hydoxy-3',4'-dioxymethylene-4-methoxymethoxy-5-(3,3-dimethyl)allylchalcone* (**6e**): Reagents: compound **5b** (500.3 mg, 1.90 mmol), 3,4-dimethoxymethoxybenzaldehyde (298.5 mg, 1.99 mmol). Eluent: petroleum ether-ethyl acetate (20:1, v/v). Product: yellow oil (540.3 mg, 72%). ^1^H-NMR (CDCl_3_, δ): 1.77 (s, 3H,), 1.78 (s, 3H), 3.31 (d, 2H, *J* = 6.8 Hz), 3.50 (s, 3H), 5.26 (s, 2H), 5.30 (m, 1H), 6.06 (s, 2H), 6.66 (s, 1H), 6.88 (d, 1H, *J* = 8.4 Hz), 7.15 (d, 1H, *J* = 8.4 Hz), 7.18 (d, 1H, *J* = 1.2 Hz), 7.40 (d, 1H, *J* = 16.0 Hz), 7.62 (s, 1H), 7.81 (d, 1H, *J* = 16.0 Hz), 13.30 (s, 1H, OH). ESI-MS: *m/z* [M+H]^+^ 397.

*2-Hydoxy-3-(1,1-methyl)allyl-4'-methoxy-5-methoxymethoxychalcone* (**6f**): Reagents: compound **5c** (500.1 mg, 1.89 mmol), 4-methoxybenzaldehyde (270.5 mg, 1.99 mmol). Eluent: petroleum ether- ethyl acetate (25:1, v/v). Product: yellow oil (180.9 mg, 25%). ^1^H-NMR (CDCl_3_, δ): 1.52 (s, 3H), 1.57 (s, 3H), 3.52 (s, 3H), 3.86 (s, 3H), 5.01 (d, 1H, *J* = 18.0 Hz), 5.02 (d, 1H, *J* = 9.6 Hz), 5.15 (s, 2H), 6.26 (dd, *J* = 18.0, 9.6 Hz), 6.94 (d, 2H, *J* = 8.4 Hz), 7.25 (d, 1H, *J* = 2.0 Hz), 7.46 (d, 1H, *J* = 2.0 Hz), 7.47 (d, 1H, *J* = 15.6 Hz), 7.61 (d, 2H, *J* = 8.4 Hz), 7.88 (d, 1H, *J* = 15.6 Hz), 13.40 (s, 1H, OH). ESI-MS: *m/z* [M+H]^+^ 383.

*2-Hydoxy-3-(1,1-methyl)allyl-4'-chloro-5-methoxymethoxychalcone* (**6g**): Reagents: compound **5c** (500 mg, 1.89 mmol), 4-chlorobenzaldehyde (279.4 mg, 1.99 mmol). Eluent: petroleum ether-ethyl acetate (25:1, v/v). Product: yellow oil (219.6 mg, 30%). ^1^H-NMR (CDCl_3_, δ): 1.52 (s, 3H), 1.55 (s, 3H), 3.51 (s, 3H), 5.01 (d, 1H, *J* = 17.2 Hz), 5.03 (d, 1H, *J* = 9.2 Hz), 5.15 (s, 2H), 6.26 (dd, *J* = 17.2, 9.2 Hz), 7.28 (d, 1H, *J* = 2.0 Hz), 7.40 (d, 2H, *J* = 8.4 Hz), 7.45 (d, 1H, *J* = 2.0 Hz), 7.55 (d, 1H, *J* = 15.6 Hz), 7.58 (d, 2H, *J* = 8.4 Hz), 7.83 (d, 1H, *J* = 15.6 Hz), 13.6 (s, 1H, OH). ESI-MS: *m/z* [M+H]^+^ 387.

### 4.4. General Method for Synthesis of Prenylated Flavanones ***7***

A solution of **6** and sodium acetate (500 mg, 6.10 mmol) in ethanol (5 mL) containing 3 drops of water was refluxed for 24 h. The mixture was poured into cold water (30 mL) and extracted with ethyl acetate (10 mL × 3 times). The organic phase was washed with brine, dried over sodium sulfate. After removing the solvent, the residue was purified by column chromatography on silica gel using petroleum ether-ethyl acetate as eluent to give **7**.

*2'-Chloro-5,7-dimethoxymethoxy-6-(3,3-dimethyl)allylflavanone* (**7a**): Reagents: compound **6a** (300 mg, 0.67 mmol); Eluent: petroleum ether-ethyl acetate (15:1, v/v). Product: pale yellow syrup (162 mg, 54%). ^1^H-NMR (CDCl_3_, *δ*): 1.66 (s, 3H), 1.69 (s, 3H), 2.75 (dd, 1H, *J* = 16.4 Hz, 2.8 Hz), 2.93 (dd, 1H, *J* = 13.4, 16.4 Hz), 3.36 (d, 2H, *J* = 6.8 Hz), 3.48 (s, 3H), 3.52 (s, 3H), 5.17 (m, 1H), 5.20 (s, 2H), 5.23 (s, 2H), 5.68 (dd, 1H, *J* = 2.8, 13.4 Hz), 6.65 (s, 1H), 7.30 (td, 1H, *J* = 2.0, 7.6 Hz), 7.36 (td, 1H, *J* = 2.0, 7.6 Hz), 7.39 (dd, 1H, *J* =2.0, 7.6 Hz), 7.20 ( dd, 1H, *J* =2.0, 7.6 Hz). ESI-MS: *m/z* [M+H]^+^ 447.

*3',4'-Dioxymethylene-5,7-dimethoxymethoxy-6-(3,3-dimethyl)allylflavanone* (**7b**): Reagents: compound **6b** (300.2 mg, 0.66 mmol). Eluent: petroleum ether-ethyl acetate (15:1, v/v). Product: pale yellow syrup (204.1 mg, 68%). ^1^H-NMR (CDCl_3_, δ): 1.71 (s, 3H), 1.73 (s, 3H), 2.76 (dd, 1H, *J* = 16.8 Hz, 2.8 Hz), 2.98 (dd, 1H, *J* = 12.8, 16.4 Hz), 3.27 (d, 2H, *J* = 7.2 Hz), 3.42 (s, 3H), 3.47 (s, 3H), 5.22 (s, 2H), 5.26 (m, 1H), 5.27 (s, 2H), 5.34 (dd, *J* = 2.8, 12.8Hz), 5.99 (s, 2H), 6.69 (s, 1H), 6.83 (d, 1H, *J* = 8.0 Hz), 6.90 (dd, 1H, *J* = 2.0, 8.0), 6.98 (d, 1H, *J* = 2.0 Hz), 7.69 (s, 1H). ESI-MS: *m/z* [M+H]^+^ 457.

*4',5,7-Trimethoxymethoxy-6-(3,3-dimethyl)allylflavanone* (**7d**): Reagent: compound **6d** (300.5 mg, 0.64 mmol). Eluent: petroleum ether-ethyl acetate (15:1, v/v). Product: pale yellow syrup (156.3 mg, 52%). ^1^H-NMR (CDCl_3_, δ): 1.68 (s, 3H), 1.70 (s, 3H), 2.82 (dd, 1H, *J* = 16.4 Hz, 2.8 Hz), 2.98 (dd, 1H, *J* =13.4, 16.4 Hz), 3.33 (d, 2H, *J* = 7.2 Hz), 3.48 (s, 3H), 3.50 (s, 3H), 3.54 (s, 3H), 5.20 (m, 1H), 5.22 (s, 2H), 5.23 (s, 2H), 5.26 (s, 2H), 5.37 (dd, 1H, *J* = 2.8 Hz, 13.4 Hz), 6.68 (s, 1H), 7.10 (d, 2H, *J* = 8.0 Hz), 7.39 (d, 2H, *J* = 8.0 Hz). ESI-MS: *m/z* [M+H]^+^ 473.

*3',4'-Dioxymethylene-5-methoxymethoxy-6-(3,3-dimethyl)allylflavanone* (**7e**): Reagents: compound **6e **(300.2 mg, 0.76 mmol). Elent: petroleum ether-ethyl acetate (12:1, v/v). Product: pale yellow syrup (180.1 mg, 60%). ^1^H-NMR (CDCl_3_, δ): 1.71 (s, 3H), 1.73 (s, 3H), 2.76 (dd, 1H, *J* = 16.8 Hz, 2.8 Hz), 2.98 (dd, 1H, *J* =12.8, 16.4 Hz), 3.27 (d, 2H, *J* = 7.2 Hz), 3.47 (s, 3H), 5.22 (s, 2H), 5.26 (m, 1H), 5.34 (dd, *J* = 2.8, 12.8Hz), 5.99 (s, 2H), 6.69 (s, 1H), 6.83 (d, 1H, *J* = 8.0 Hz), 6.90 (dd, 1H, *J* = 2.0, 8.0), 6.98 (d, 1H, *J* = 2.0 Hz), 7.69 (s, 1H). ESI-MS: *m/z* [M+H]^+^ 397.

*4'-Methoxy-6-methoxymethoxy-8-(1,1-dimethyl)allyl-flavanone* (**7f**): Reagent: compound **6f** (300.0 mg, 0.79 mmol). Elu-nt: petroleum ether-ethyl acetate (15:1, v/v). Product: pale yellow syrup (150 mg, 50%). ^1^H-NMR (CDCl_3_, δ): 1.41 (s, 3H), 1.45 (s, 3H), 2.85 (dd, *J* = 2.0, 16.8 Hz, 1H), 3.02 (dd, *J* = 13.2, 16.8 Hz, 1H), 3.48 (s, 3H), 3.92 (s, 3H), 4.94 (d, 1H, *J* = 17.2 Hz), 4.95 (d, 1H, *J* = 10.8 Hz), 5.21 (s, 2H), 5.24 (dd, *J* = 2.0, 13.2 Hz), 6.09 (dd, 1H, *J* = 10.8, 17.2 Hz), 7.01 (d, 2H, *J* = 8.4 Hz), 7.12 (d, 1H, *J* = 2.4 Hz), 7.38 (d, 1H, *J* = 2.4 Hz), 7.40 (d, 2H, *J* = 8.4 Hz). ESI-MS: *m/z* [M+H]^+^ 383.

*4'-Chloro-6-methoxymethoxy-8-(1,1-dimethyl)allyl-flavanone* (**7g**): Reagents: compound **6g** (300.3 mg, 0.78 mmol). Eluent: petroleum ether-ethyl acetate (15:1, v/v). Product: pale yellow syrup (129.1 mg, 43%). ^1^H-NMR (CDCl_3_ δ): 1.42 (s, 3H), 1.48 (s, 3H), 2.83 (dd, 1H, *J* = 2.0, 16.8 Hz), 3.95 (dd, 1H, *J* = 13.2, 16.8 Hz), 3.44 (s, 3H), 4.90 (d, 1H, *J* = 17.2 Hz), 4.98 (d, 1H, *J* = 10.8 Hz), 5.25 (s, 2H), 5.32 (dd, 1H, *J* = 2.0, 13.2 Hz), 6.03 (dd, 1H, *J* = 10.8, 17.2 Hz), 7.13 (d, 1H, *J* = 2.4 Hz), 7.39 (d, 1H, *J* = 2.4 Hz), 7.44 (brd, 4H). ESI-MS: *m/z* [M+H]^+^ 387.

### 4.5. General Method for Synthesis of Compounds ***10***

A solution of compound **8**, 2-chloro-diethylethanamine hydrochloride and potassium carbonate in acetone (5 mL) was refluxed for 10 h. After cooling to room temperature, the mixture was filtered and the solution was concentrated *in vacuo.* The residue was purified by column chromatography on silica gel using petroleum ether-ethyl acetate as eluant (3:1, v/v) to give **9**, which was further dissolved in 5 mL 3N HCl/methanol (1/4, v/v) and refluxed for 1 h. Then, the reaction mixture was evaporated *in vacuo* to give yellow solid and washed with ether to afford **10**.

*2-(2-(Diethylamino)ethoxy-3'-bromo-4,6-dimethoxymethoxychalcone* (**10a**): Reagents: compound **8a **(200 mg, 0.47 mmol), 2-chloro-*N,N*-diethylethanamine hydrochlororide (122 mg, 0.71 mmol) and potassium carbonate (194.6 mg, 1.41 mmol). Product: yellow solid (167.3 mg, 72%), m.p. 180–182 °C. ^1^H-NMR (MeOH-d_4_, *δ*): 1.08 (d, 1H, *J* = 6.8 Hz), 3.10 (m, 4H), 3.48 (m, 2H), 4.38 (brs, 2H), 6.03 (s, 1H), 6.07 (s, 1H), 7.40 (d, 1H, *J* = 8.0 Hz), 7.51 (d, 1H, *J* = 16.0 Hz), 7.63 (d, 1H, *J* = 8.0 Hz ), 7.65 (d, 1H, *J* = 16.0 Hz), 7.78 (d, 1H, *J* = 8.0 Hz), 8.01 (s, 1H), 10.06 (s, 1H, OH), 10.37 (s, 1H, OH), 12.48 (s, 1H, OH). ESI-MS: *m/z* [M+H]^+^ 434.

*2-(2-(Diethylamino)ethoxy-3',4'-dioxymethylene-4,6-dimethoxymethoxy-chalcone* (**10b**): Reagents: compound **8b** (201 mg, 0.52 mmol), 2-chloro-*N,N*-diethylethanamine hydrochlororide (133.7 mg, 0.78 mmol) and potassium carbonate (215.3 mg, 1.56 mmol). Product: yellow solid (161.8 mg, 78%), m.p. 227–230 °C. ^1^H-NMR (MeOH-d_4_, *δ*): 1.10 (d, 1H, *J* = 6.8 Hz), 3.10 (m, 4H), 3.49 (m, 2H), 4.39 (brs, 2H), 6.02 (s, 1H), 6.06 (s, 1H), 6.10 (s, 2H), 6.97 (d, 1H, *J* = 8.0 Hz), 7.25 (d, 1H, *J* = 8.0 Hz), 7.46 (s, 1H), 7.45 (d, 1H, *J* = 16.0 Hz), 7.51 (d, 1H, *J* = 16.0 Hz), 10.07 (s, 1H, OH), 10.55 (s, 1H, OH), 12.55 (s, 1H, OH). ESI-MS: *m/z* [M+H]^+^ 400.

### 4.6. General Method for Synthesis of Compounds ***11***

A solution of **7** in 3N HCl/methanol (1/4, v/v, 3 mL) was refluxed for 2 h, then poured into cold water (15 mL) and extracted with ethyl acetate (5 mL × 3 times). The organic phase was washed with brine and then dried over anhydrous sodium sulfate. After removal of the solvent, the residue was purified by column chromatography on silica gel using petroleum ether-ethyl acetate as eluant to give **11**.

*2'-Chloro-5,7-dihydroxy-6-(3,3-dimethyallyl)flavanone* (**11a**): Reagents: compound **11a** (150 mg, 0.34 mmol). Eluent: petroleum ether-ethyl acetate (6:1, v/v). Product: white solid (90.3 mg, 75%), m.p. 130–133 °C. ^1^H-NMR (acetone-d_6_, δ): 1.59 (s, 3H), 1.69 (s, 3H), 2.78 (dd, 1H, *J* = 2.8, 17.2 Hz), 3.07 (dd, 1H, *J* = 12.8, 17.2 Hz), 3.12 (d, 2H, *J* = 6.8 Hz), 5.17 (m, 1H), 5.79 (dd, 1H, *J* = 2.8, 12.8 Hz), 5.99 (s, 1H), 7.39–7.48 (m, 3H), 7.77 (dd, 1H, *J* =1.6, 7.6 Hz), 9.66 (s, 1H), 12.35 (s, 1H). ESI-MS: *m/z* [M+H]^+^ 359.

*3',4'-Dioxymethylene-5,7-dihydroxy-6-(3,3-dimethyl)allylflavanone* (**11b**): Reagents: compound **11b** (150 mg, 0.33 mmol). Eluent: petroleum ether-ethyl acetate (6:1, v/v). Product: white solid (79.9 mg, 68%), m.p. 156–158 °C. ^1^H-NMR (acetone-d_6_, δ): 1.59 (s, 3H), 1.68 (s, 3H), 2.69 (dd, *J* =3.2, 16.8 Hz, 1H), 3.10 (dd, 1H, *J* = 12.8, 16.8 Hz), 3.20 (d, 2H, *J* = 7.2 Hz), 5.18 (m, 1H), 5.39 (dd, 1H, *J* = 12.8 Hz, 3.2 Hz), 5.98 (s, 2H), 6.00 (s, 1H), 6.83 (d, 1H, *J* = 8.0 Hz), 6.97 (d, *J* = 8.0 Hz), 7.04 (s, 1H), 9.53 (s, 1H), 12.39 (s, 1H). ESI-MS: *m/z* [M+H]^+^ 369.

*2-(4'-Hydroxyphenyl)-5-hydroxy-6,7-dihydro-8,8-dimethyl-4H,8H-benzo[1,2-b;5,4-b']dipyran-4-one* (**11d**): Reagents: compound **11d** (150 mg, 0.32 mmol). Eluent: petroleum ether-ethyl acetate (6:1, v/v). Product: white solid (41.1 mg, 38%), m.p. 122–125 °C. ^1^H-NMR (DMSO-d_6_, *δ*): 1.33 (s, 6H), 1.82 (t, 2H, *J* = 7.2 Hz), 2.58 (t, 2H, *J* = 7.2 Hz), 2.73 (dd, 1H, *J* = 2.8, 17.2 Hz), 3.16 (dd, 1H, *J* = 12.8, 17.2 Hz), 5.42 (dd, 1H, *J* = 2.8, 12.8 Hz), 5.84 (s, 1H), 6.89 (d, 1H, *J* = 8.0 Hz), 7.38 (dd, 1H, *J* = 8.0 Hz), 8.51 (1H, OH), 12.57 (1H, OH). ESI-MS: *m/z* [M+H]^+^ 341.

*2-(3,4-Dioxymethylenephenyl)-6,7-dihydro-8,8-dimethyl-4H,8H-benzo[1,2-b;5,4-b']dipyran-4-one* (**11e**): Reagents: compound **11e** (150 mg, 0.38 mmol). Eluent: petroleum ether: ethyl acetate (6:1, v/v). Product: white solid (60 mg, 45%), m.p. 95–98 °C .^1^H-NMR (DMSO-d_6_, *δ*): 1.34 (s, 3H), 1.35 (s, 3H), 1.81 (t, 1H, *J* = 6.8 Hz), 2.76 (t, 1H, *J* = 6.8 Hz), 2.77 (dd, 1H, *J* = 2.4, 17.2 Hz), 2.98 (dd, 1H, *J* = 12.8, 17.2 Hz), 5.32 (dd, 1H, *J* = 2.4, 12.8 Hz), 5.97 (s, 2H), 6.38 (s, 1H), 6.82 (d, 1H, *J* = 7.6 Hz), 6.90 (dd, 1H, *J* = 2.0, 7.6 Hz), 6.97 (d, 1H 1H, *J* = 2.0 Hz), 7.26 (s, 1H), 7.67 (s, 1H). ESI-MS: *m/z* [M+H]^+^ 353.

*4'-Methoxy-6-hydroxy-8-(1,1-dimethyl)allyl-flavanone* (**11f**): Reagents: compound **11f** (150 mg, 0.39 mmol). Eluent: petroleum ether: ethyl acetate (10:1, v/v). Product: yellow syrup (55.7, 42%). ^1^H-NMR (CDCl_3_ δ): 1.43 (s, 3H), 1.44 (s, 3H), 2.82 (dd, *J* = 2.0, 16.8 Hz, 1H), 3.00 (dd, *J* = 13.2, 16.8 Hz, 1H), 3.84 (s, 3H), 4.89 (d, 1H, *J* = 17.2 Hz), 4.95 (d, 1H, *J* = 10.8 Hz), 4.97 (s, 1H, OH), 5.30 (dd, *J* = 2.0, 13.2 Hz), 6.11 (dd, 1H, *J* = 10.8, 17.2 Hz), 6.95 (d, 2H, *J* = 8.4 Hz), 7.11 (d, 1H, *J* = 2.4 Hz), 7.27 (d, 1H, *J* = 2.4 Hz), 7.40 (d, 2H, *J* = 8.4 Hz). ESI-MS: *m/z* [M+H]^+^ 339.

*4'-Chloro-6-hydroxy-8-(1,1-dimethyl)allyl-flavanone* (**11g**): Reagents: compound **11g** (100 mg, 0.26 mmol). Eluent: petroleum ether-ethyl acetate (10:1, v/v). Product: yellow solid (29.2 mg, 33%), m.p. 90–92 °C. ^1^H-NMR (CDCl_3_ δ): 1.42 (s, 3H), 1.45 (s, 3H), 2.83 (dd, 1H, *J* = 2.0, 16.8 Hz), 3.92 (dd, 1H, *J* = 13.2, 16.8 Hz), 4.89 (d, 1H, *J* = 17.2 Hz), 4.95 (d, 1H, *J* = 10.8 Hz), 5.00 (s, 1H, OH), 5.30 (dd, 1H, *J* = 2.0, 13.2 Hz), 6.09 (dd, 1H, *J* = 10.8, 17.2 Hz), 7.13 (d, 1H, *J* = 2.4 Hz), 7.36 (d, 1H, *J* = 2.4 H), 7.41 (brd, 4H). ESI-MS: *m/z* [M+H]^+^ 343.

### 4.7. Vasorelaxant Activities Assay

Vascular rings were prepared from the aorta of male Male Sprague-Dawley rats (four to six months old and weighing on average 250 g), and contraction studies were performed following the general procedure detailed in the literature [18]. After an equilibration period of at least 1 h, isometric contractions induced by PE (1 μM) were obtained. When contraction of the tissue in response to this vasoconstrictor agent had stabilized (after about 20 min), cumulatively increasing concentrations of the tested compounds were added to the bath at 15~20 min intervals (the time needed to obtain steady-state relaxation). Control tissues were simultaneously subjected to the same procedures, but omitting the compounds and adding the vehicle. The flavonoids-induced maximal relaxation (*E_max_*) in aortic rings was calculated as a percentage of the contraction in response to PE (1 μM). The half maximum effective concentration (EC_50_) was defined as the concentration of the flavonoids that induced 50% of maximum relaxation from the contraction elicited by PE (1 μM) and was calculated from the concentration response curve by nonlinear regression (curve fit) using GraphPad Prism (Version 4.0).

### 4.8. Computational Methods

#### 4.8.1. Data Preparation

For the development of 2D-QSAR model, five newly synthesized compounds and thirty previous compounds [14,15,16,19] with available EC_50_ were selected. They were divided into training set (26 molecules) and test set (nine molecules) considering both structural diversity and wide coverage of biological activity ranges. The structures of all compounds were optimized in Discovery Studio 2.0 software (Accelrys, Inc. San Diego, CA, USA). The resulted geometry of molecules were inputted into Dragon software [20], which can calculate constitutional descriptors, topological descriptors, walk and path counts, information indices, 2D autocorrelations, edge adjacency indices, Burden eigenvalue descriptors, Burden eigenvalue descriptors, *etc.* After the calculation of the molecular descriptors, those that stayed constant for all molecules were eliminated, and pairs of variables with a correlation coefficient greater than 0.75 were classified as intercorrelated with one of each correlated pair was deleted.

#### 4.8.2. Enhanced Replacement Method-Multiple Linear Regressions (ERM-MLR)

Enhanced Replacement Method (ERM) proposed by Mercader *et al.* [21] is a modified version of Replacement Method (RM) [22,23]. The purpose of both algorithms is to choose an optimal subset of d (d < D) descriptors from the pool of D descriptors with minimum standard deviation SD:

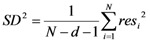

where N is the number of molecules in the training set, and *res_i_* the residual for molecule *i* (difference between the experimental and predicted property). Considering that SD(d_n_) is a distribution on a discrete space of D!/d!(D–d) disordered points d*_n_*, ERM produces linear models that are quite similar with the full search (FS) but with much less computational work. First, an initial set of descriptors *d_k_* was selected at random. And one of the descriptors, say *X_ki_*, with all the remaining descriptors (D-d) was replaced by other descriptor, one by one, and the set with the smallest value of SD was kept. Second, from the resulting set the descriptor with the greatest SD in its coefficient is chosen and all the remaining D-d descriptors, one by one, until the set remains unmodified. More detailed information about these algorithms can be found in reference [21].

#### 4.8.3. Leave-One-Out Cross Validation

Validation of the models was required to test the predictive ability and generalization of the methods by cross validation as well as test set prediction. The leave-one-out cross validation, a special case of the cross-validation technique [24] was employed to find the promising QSAR model. Given n samples available in a data set and m candidate models, each model is trained with n − 1 samples and then is tested on the sample that was left out. This process is repeated n times until every sample in the data set have been used once as a cross-validation instance. Finally, cross validation correlation coefficient (*Q_loo_^2^*) of LOO-CV, a measure of the model generalization capability, for all candidate models can be obtained as below:

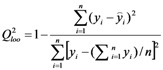

where *y_i_* is the desired output and 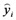 is the actual output of the model, and *n* is the number of compounds in the analyzed set.
